# ASSESSING PREFERENCES FOR COMMUNICATING WITH TECHNOLOGY: COMMUNITY-BASED PROVIDER PERSPECTIVES

**DOI:** 10.1093/geroni/igz038.3334

**Published:** 2019-11-08

**Authors:** Andrea Y Sillner, Kimberly VanHaitsma, Rachel Wion, Marie Boltz

**Affiliations:** The Pennsylvania State University, University Park, Pennsylvania, United States

## Abstract

Miscommunication during older adults’ care transitions from hospital to community-based settings (e.g. home health) can lead to adverse events. Effective use of technology assisted communication (TAC) may help to remedy miscommunication surrounding care transitions. Care providers in community-based settings are well-positioned to provide insight on the feasibility and current use of TAC. The purpose of this research was to determine contextual factors (i.e., intrapersonal, interpersonal, environmental) that influence the use of TAC in the home health setting from the perspective of community-based direct care providers and administrators. Focus groups were conducted with direct care providers and c administrators from two different settings – rural and urban/suburban. Content analysis was used to determine themes. Participants indicated that there are many barriers for older adults’ use of TAC such as low interest, fear of technology, knowledge gaps, and lack of access to technology. However, others embraced the use of TAC and technology in the community-based care. Additionally, certain forms of TAC, such as text and email, may be better for communicating with informal caregivers. Some direct care providers indicated they were not allowed or encouraged to use certain TAC with patients due to potential security concerns. The community-based care administrators highlighted the importance of TAC but did indicate that use can be limited due to liability and HIPAA concerns. These findings provide important insight for both determining how to best implement TAC for older adults in community-based care settings and aiding in the development of a tool for measuring preferences.

